# Ultrafast Laser-Excited Optical Emission of Xe under Loose-Focusing Conditions

**DOI:** 10.3390/s23239374

**Published:** 2023-11-23

**Authors:** Miloš Burger, Kyle S. Latty, Leandro Frigerio, Thiago Arnaud, Kyle C. Hartig, Igor Jovanovic

**Affiliations:** 1 Department of Nuclear Engineering and Radiological Sciences, University of Michigan, Ann Arbor, MI 48109, USA; leandrof@umich.edu (L.F.);; 2 Gérard Mourou Center for Ultrafast Optical Science, University of Michigan, Ann Arbor, MI 48109, USA; 3 Nuclear Engineering Program, Department of Materials Science and Engineering, University of Florida, Gainesville, FL 32611, USA; klatty@ufl.edu (K.S.L.);

**Keywords:** ultrafast nonlinear optics, self-focusing, atomic emission spectroscopy

## Abstract

The optical filament-based radioxenon sensing can potentially overcome the constraints of conventional detection techniques that are relevant for nuclear security applications. This study investigates the spectral signatures of pure xenon (Xe) when excited by ultrafast laser filaments at near-atmosphericpressure and in short and loose-focusing conditions. The two focusing conditions lead to laser intensity differences of several orders of magnitude and different plasma transient behavior. The gaseous sample was excited at atmospheric pressure using ∼7 mJ pulses with a 35 fs pulse duration at 800 nm wavelength. The optical signatures were studied by time-resolved spectrometry and imaging in orthogonal light collection configurations in the ∼400 nm (VIS) and ∼800 nm (NIR) spectral regions. The most prominent spectral lines of atomic Xe are observable in both focusing conditions. An on-axis light collection from an atmospheric air–Xe plasma mixture demonstrates the potential of femtosecond filamentation for the remote sensing of noble gases.

## 1. Introduction

The detection of trace concentrations of Xe has seen an increased interest in nuclear safety and security [1,2]. For example, Xe can be traced as an indicator of fuel failure in light water reactors for off-gas sensing in molten salt reactors [3] and as an early indicator of fuel failure in the coolant loop of the proposed generation IV gas-cooled fast reactors (GCFRs) [4,5]. Being one of the most prominent and mobile gaseous fission products, the occurrence of Xe and its isotopes can provide temporal and compositional information on the release following a nuclear detonation or an accident occurring at a nuclear facility [6,7]. The sensitive detection of various Xe isotopes at standoff distances has been employed to detect nuclear detonations and is an important method for verification of the Comprehensive Test Ban Treaty [8]. The remote detection of Xe could address challenges that arise in responding to radioactive releases and contamination where hazardous exposure levels are present.

The conventional methods for discerning and measuring nuclear substances, encompassing both radiological material and special nuclear material, are based on the detection of ionizing radiation generated during radioactive decay. Pertinent radiation characteristics comprise subatomic particles such as α and β particles, as well as neutrons, in addition to electromagnetic radiation, such as gamma rays and X-rays. Comparable to atomic spectroscopy, the energy contained within emitted radiation often encodes distinctive details about the originating nuclide. However, ionizing radiation exhibits a restricted penetration depth when traversing through media such as air. When it comes to tracking radioxenon isotopes in the atmosphere, the identification of collected xenon samples is typically carried out through one of two methods: (*i*) β–γ coincidence spectrometry using scintillators [9] or (*ii*) high-resolution gamma-ray spectrometry [10].

As an alternative, laser-induced breakdown spectroscopy (LIBS) is a common method for the generation of material-specific optical signatures [11]. It is a candidate for practical, remote detection and the tracking of Xe that does not rely on radioactive decay. In LIBS, a high-power pulsed laser ionizes the target sample into a plasma; spectrally and temporally resolved emission from the plasma then provides information on its constituent elements and their respective concentrations. In our previous work, we used nanosecond LIBS to detect trace quantities of Xe mixed with a He buffer gas, which simulates the conditions encountered in GCFR coolant monitoring. In these experiments, we have shown that near-real-time detection with µmol mol${}^{-1}$ Xe sensitivity can be realized in a relatively simple single-pulse LIBS measurement [12,13]. When compared to nanosecond LIBS, femtosecond (fs) LIBS offers several benefits, including reduced continuum radiation, sample damage, and susceptibility to sample matrix and fractionation effects [14,15]. Moreover, fs LIBS offers a path to measure and analyze samples over long distances through the process of laser filamentation [16]. When the fs laser power is higher than the critical power $P_{cr}$ for self-focusing in a given medium, a laser filament is generated that supports extended laser propagation at high intensities without the use of an external focusing optic. In fs laser filamentation, an interplay between the beam focusing, due to the nonlinear Kerr effect, and defocusing, due to the generation of plasma and diffraction, takes place [17]. Even in a low-density medium such as the air, millijoule-level pulses can support the formation of filaments that extend over several meters. The confinement of laser power in a small beam through filamentation enables the ablation of solid targets and plasma generation over long distances even as the fraction of the laser energy is expended to generate the plasma column in the air [18,19]. Early investigations have shown signal detection at ranges of up to 180 m [20], and they have solidified free-propagating femtosecond filamentation as a promising technique for making filament-LIBS (FIBS) a practical possibility. In this strategy, the second-order dispersion, often referred to as chirp, is fine-tuned to counteract the effects of air dispersion. For instance, the dispersion control enables the creation of a filament at a specified distance and can be relevant in remote nuclear material sensing [21]. Furthermore, past efforts have demonstrated uranium isotopic differentiation from a standoff distance [22] by using the femtosecond filamentation-laser ablation molecular isotopic spectrometry (F2-LAMIS) technique. Although successful, most previous demonstrations were limited to solid targets [23].

Filament-based techniques for gas and aerosol sensing have also been shown, focusing on the quantification of atmospheric pollutants, monitoring chemical reactions such as combustion, or identifying bio-hazards [24,25,26]. This family of techniques includes several types of filament-based sensing methods, such as filament-induced plasma spectroscopy [27], filamentation-based white-light LIDAR [28], filamentation-assisted terahertz remote sensing [29], and filament-driven impulsive Raman spectroscopy [30,31]. All of these methods make use of the unique properties of filaments, including self-compression, plasma-induced fluorescence, white-light generation, and terahertz wave generation [32]. Regarding the detection of atmospheric pollutants, the use of novel algorithms for data processing can enhance the detection limits of filament-induced plasma spectroscopy; for example, the detection of 1.4 µg/m${}^{3}$ of Na${}^{+}$ in an aerosol at 30 m of distance was reported recently [33]. In multi-component gas detection applications, fluorescence from dissociated methane and acetylene radicals has been used to detect concentrations of 1 part per million (ppm) and 300 parts per billion (ppb), respectively. In the same study, a genetic algorithm has been used to infer the absolute concentration of each component with an error of 25% for unknown samples [34].

The physical basis for improving signals from filaments has been studied. For example, the backward stimulated emission of N${}_{2}$ from filaments has been reported in on-axis configuration with a strong dependence on the polarization state [35]. Also, the enhancement of the fluorescent signal up to 16 times has been achieved using a second ns pulse superimposed on the filament [36]. Filamentation has also been used to induce the laser-induced fluorescence of UO${}_{2}$F${}_{2}$ solution [37]. Furthermore, a technique based on a femtosecond laser and plasma spectroscopy was used to measure the isotopic composition of UF${}_{6}$ using optical signatures in the 424.4 nm spectral region [38]. The results were in good agreement with the actual isotopic composition of the samples, reporting values within 0.28% of the actual ${}^{235}$U content of the samples and a standard deviation of 0.90%.

However, with LIBS, challenges remain with the ability to separate Xe isotope signatures. Depending on the plasma temperature, Doppler broadening can play a significant role in the later stages of plasma lifetime. Also, due to the large mass of Xe, the isotope shifts that are on the order of pm or smaller [39,40] are challenging to measure even with high-resolution spectrometers and photomultiplier detectors; the use of an interferometric approach is required. Nevertheless, the existing literature lacks not only work on filamentation-based gas sensing for nuclear applications but also investigations of atomic gas signatures that can be detected using this powerful technique [32,41].

Here, we explore the spectral emission of pure Xe at near atmospheric pressure excited by focused femtosecond laser pulses and assess the viability of using short-lived ultrafast laser filaments for Xe excitation. Time-resolved imaging is used with the optimization of the gate delay and gate width to demonstrate the temporal evolution of the Xe spectra. We show that the intensity produced and maintained as the beam undergoes femtosecond filamentation is sufficient to excite many VIS and NIR transitions. Furthermore, our on-axis measurements of Xe signatures in an air–Xe mixture show promise for the implementation of atomic emission spectroscopy in various practical remote gas-sensing scenarios.

## 2. Experimental Methods

**Figure 1:**
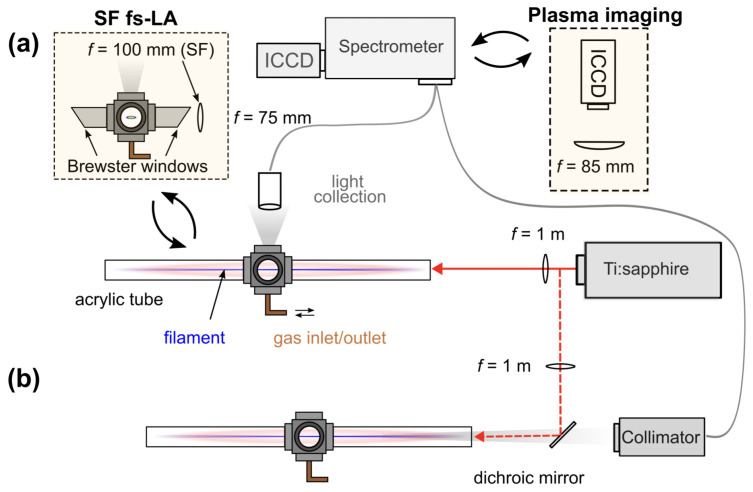
(**a**) Experimental setup for LIBS and plasma imaging for loose-focusing (filaments) and short focus fs pulses in pure Xe; (**b**) on-axis light collection setup of air-Xe mixture plasma emission.

A simplified schematic of the LIBS and plasma imaging experimental setup is shown in Figure 1. A chirped-pulse amplified Ti:sapphire laser (Coherent Astrella) was operated at a repetition rate of 1 kHz and produced ∼7 mJ pulses with a 35 fs pulse duration centered at 800 nm. A sealed gas chamber was first evacuated and subsequently filled with 99.99% purity Xe to 778 Torr for all experiments. Both the plasma imaging and spectroscopy were performed orthogonal to the beam path through a side-viewing window. Time-resolved spectroscopy was performed using a Czerny–Turner spectrometer (Princeton Instruments HRS-500, New Jersey, USA) with an electron-multiplying intensified CCD (emICCD, Princeton Instruments PI-MAX4) using an f=75 mm and d=25.4 mm collection lens. The spectrometer slit width was set to 100 µm. Images were recorded with an intensified CCD (ICCD, Andor iStar 334T) using a f=85.2 mm and d=76.2 mm imaging lens adjusted to result in a ∼2× magnification. A 1 m focusing lens was used to generate filaments, corresponding to a loose-focus configuration. Acrylic tubes with thin laser windows were attached to the gas chamber as shown in Figure 1a to extend the enclosed space closer to the focusing lens, where the spot size is sufficiently large to avoid the significant accumulation of a nonlinear phase and optical damage. The light collection was optimized by observing the first negative band ($B^{2}\Sigma_{u}^{+}\to X^{2}\Sigma_{g}^{+}$) of $N_{2}^{+}$ under ambient air conditions. Under short-focus conditions, a 100 mm lens was used for comparison with the loose-focus configuration. When focusing the beam with a 100 mm lens, filamentation is not observed, and thus, the intensity is not clamped as in the case of a filament [42].

To mimic a practical remote sensing configuration, an on-axis collection of filament-induced air–Xe fluorescence was performed under atmospheric pressure as shown in Figure 1b. The gas cell was evacuated using a mechanical pump before introducing air and Xe. The Xe fraction in the gas mixture was set to 2% (partial pressure). The laser beam was diverted after the focusing lens using a 45${}^{\circ }$ dichroic mirror. In this configuration, the light collection relied on the fiber-coupled collimator (Andor CC52) positioned in line with the filamentation axis approximately 0.6 m from the filament.

## 3. Results and Discussion

The laser intensity in the short-focus case is estimated to be in the order of ∼10${}^{17}$ W cm${}^{-2}$, whereas in the loose-focus case, it is clamped to ∼10${}^{13}$ W cm${}^{-2}$ in air [17]. Several orders of magnitude difference in intensities manifests in the transient character of the plasmas that each of these two focusing geometries produces. Both focusing geometries were examined by monitoring the VIS and NIR spectral regions.

### 3.1. VIS Spectral Region

**Figure 2:**
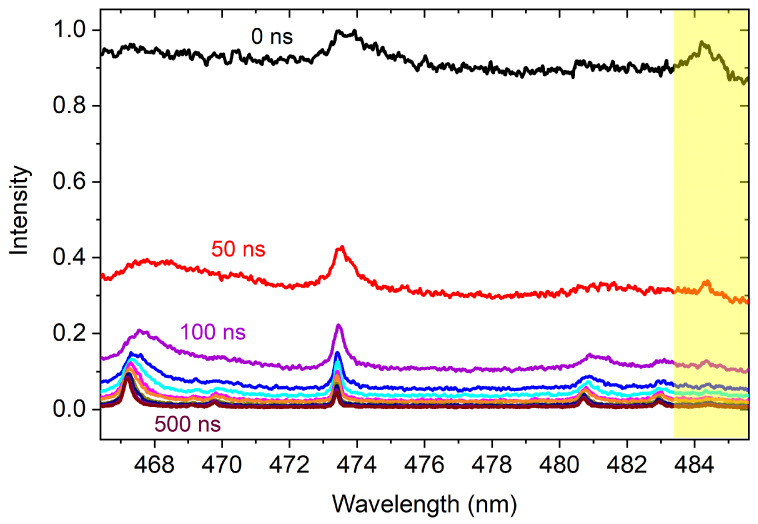
Early-stage short-focus temporal evolution of Xe spectra in the VIS region. The highlighted region indicates the presence of weak Xe II emission.

In our experiments that employed the short-focusing configuration, Xe emission is observed up to several microseconds after the laser pulse, while the continuum emission diminishes almost entirely after several hundreds of nanoseconds (Figure 2). For comparison, the strong ionic emission in the broadband VIS spectral region can be seen in our previous work using ns excitation [43]. The spectral region centered around 476 nm is known to contain a large number of Xe II transitions in the case of nanosecond excitation. However, the high-lying ionic energy states are more difficult to excite using femtosecond pulses, as evidenced by the highlighted region in Figure 2. The Xe II transition at 484.4 nm represents one of the strongest observable Xe emissions under typical nanosecond laser-produced plasma conditions [13], yet the ionic emission is barely observable and only persists for ∼100 ns in our short-focus experiments. Femtosecond pulses are more efficient in producing free carriers through multiphoton ionization during the initial phase of the pulse. Conversely, when working with nanosecond pulses, the importance of multiphoton ionization diminishes, and the breakdown primarily originates from a small number of free carriers that existed before the arrival of the pulse. This process is followed by a strong absorption of these carriers by the generated plasma, resulting in further heating and ionization [44]. While the interaction between the femtosecond laser pulse and the gas is considered non-thermal, it is anticipated that the plasma following the femtosecond laser pulse will approach local thermodynamic equilibrium owing to the intense collision rates occurring at atmospheric pressure. Given that a plasma at atmospheric pressure typically has an electron–ion relaxation time in the order of nanoseconds [45], the intensity and persistence of ionic emission are relatively weak. At later times, the spectrum is dominated by atomic transitions, whose characteristics are provided in Table 1. As the electron density decreases with time, the Stark-broadened spectral line profiles become narrower.

**Figure 3:**
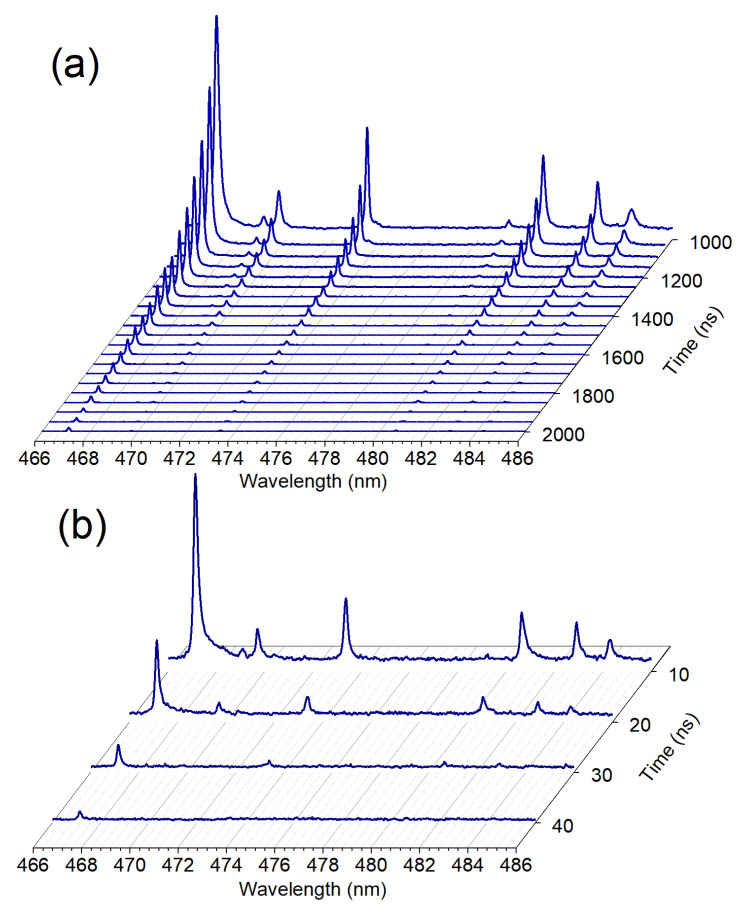
(**a**) Short-focus, late-stage; (**b**) loose-focus, early-stage temporal evolution of Xe spectra in the VIS region.

**Figure 4:**
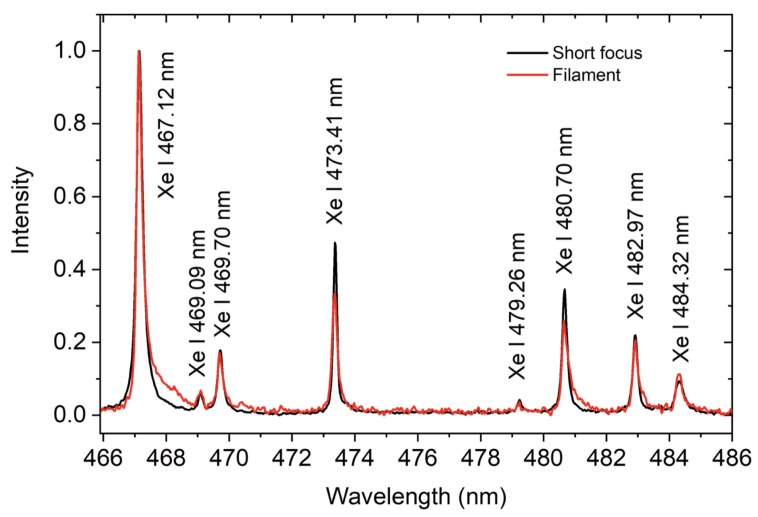
Typical spectrum of Xe I transitions in the VIS spectral range obtained using both short and loose focusing. Intensity is normalized for comparison.

The time-resolved spectra for both focusing configurations can be seen in Figure 3. Since the filament plasma does not experience similar thermalization effects like the short-focus-produced plasma, its lifetime and, therefore, optical emission persistence, is affected [46]. Unless otherwise specified, the recording gate width was 50 ns for short-focus and 10 ns for loose-focus excitation. The typical resulting spectra are a result of 30 accumulated shots for the short-focus case and up to 10${}^{4}$ for the loose-focus case. The lifetime of filament fluorescence was observed to be under 100 ns at 810 Torr of Xe (Figure 3b). After 40 ns after the laser pulse, only one Xe I spectral line centered at 467.12 nm can be observed in the case of loose-focusing configuration. All transitions present in the short-focus case are also present in the loose-focus case, as evidenced by the time-integrated spectral measurements shown in Figure 4. The small (∼15%) difference between intensities of transitions originating from the higher-lying (>11 eV) and other upper energy levels (Table 1) indicates the efficacy of filament excitation in producing characteristic spectral signatures of xenon.

**Table 1:** Wavelength, transition probability, configurations and terms of upper and lower excitation levels of Xe I transitions according to NIST [47].

Species	Wavelength	Einstein Coeff.	Lower Level		Upper Level		Lower Level	Upper Level
	**(nm)**	($10^{6}s^{-1}$)	**Config.**	**Term**	**Config.**	**Term**	**Energy (eV)**	**Energy (eV)**
Xe I	467.123	2.49	$5p^{5}6s$	${}^{2}P_{3/2}^{o}$	$5p^{5}7p$	${}^{2}P_{3/2}^{o}$	8.315	10.969
	469.097	0.28	$5p^{5}6s$	${}^{2}P_{3/2}^{o}$	$5p^{5}6p$	${}^{2}P_{1/2}^{o}$	8.315	10.958
	469.702	0.736	$5p^{5}6s$	${}^{2}P_{3/2}^{o}$	$5p^{5}7p$	${}^{2}P_{3/2}^{o}$	8.315	10.954
	473.416	1.38	$5p^{5}6s$	${}^{2}P_{3/2}^{o}$	$5p^{5}6p$	${}^{2}P_{1/2}^{o}$	8.437	11.055
	479.262	0.12	$5p^{5}6s$	${}^{2}P_{3/2}^{o}$	$5p^{5}7p$	${}^{2}P_{3/2}^{o}$	8.315	10.902
	480.702	4.29	$5p^{5}6s$	${}^{2}P_{3/2}^{o}$	$5p^{5}7p$	${}^{2}P_{3/2}^{o}$	8.437	11.015
	482.971	0.6	$5p^{5}6s$	${}^{2}P_{3/2}^{o}$	$5p^{5}7p$	${}^{2}P_{3/2}^{o}$	8.437	11.003
	484.329	0.702	$5p^{5}6s$	${}^{2}P_{3/2}^{o}$	$5p^{5}7p$	${}^{2}P_{3/2}^{o}$	8.437	10.996
	820.633	20	$5p^{5}6s$	${}^{2}P_{1/2}^{o}$	$5p^{5}6p$	${}^{2}P_{3/2}^{o}$	9.447	10.957
	823.163	28.6	$5p^{5}6s$	${}^{2}P_{3/2}^{o}$	$5p^{5}6p$	${}^{2}P_{3/2}^{o}$	8.315	9.821
	826.652	16.2	$5p^{5}6s$	${}^{2}P_{1/2}^{o}$	$5p^{5}6p$	${}^{2}P_{1/2}^{o}$	9.569	11.069
	828.011	36.9	$5p^{5}6s$	${}^{2}P_{3/2}^{o}$	$5p^{5}6p$	${}^{2}P_{3/2}^{o}$	8.436	9.933
	834.682	42	$5p^{5}6s$	${}^{2}P_{1/2}^{o}$	$5p^{5}6p$	${}^{2}P_{1/2}^{o}$	9.569	11.055

### 3.2. NIR Spectral Region

**Figure 5:**
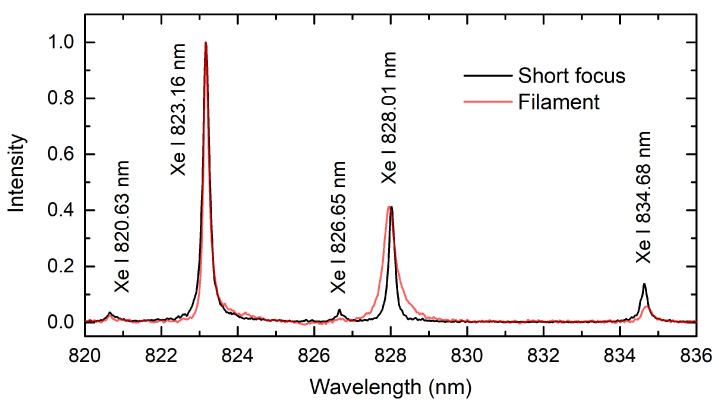
Near-IR Xe spectra obtained using short and loose-focusing configurations.

Figure 5 shows the comparison between the Xe spectra excited by short-focus and loose-focus geometries. The recording parameters were 1000 ns delay, 100 ns gate width; and 10 averaged shots for the short focusing, and 0 ns delay, 50 ns gate width, and 5000 averaged shots for the loose focusing. In contrast to the relatively strong spectral lines at 823.16 nm and 828.01 nm originating from the 9.8-eV level, the occurrence of higher-lying transitions at 820.63 nm, 826.65 nm, and 834.68 nm (>10.9 eV) is minuscule, especially in the case of filament excitation. The ratio of peak intensities remained approximately the same for both focusing geometries in Figure 5, while the line at 828.01 nm experienced notable broadening. In comparison to the short-focus case, this excessive broadening may be explained by the larger transition probability and increased self-absorption. An asymmetry in line profiles for loose-focus spectral lines indicates the non-uniform character of the plasma along the light collection axis.

**Figure 6:**
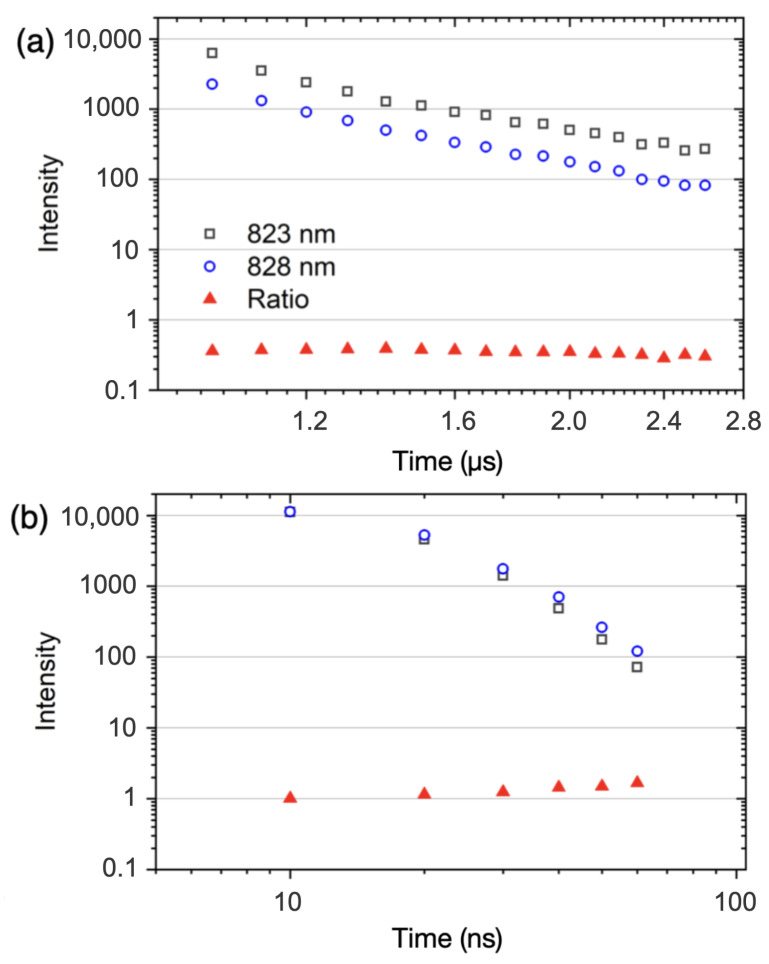
Time-resolved fs-induced Xe emission intensity for atomic spectral lines at 823.16 nm and 828.01 nm using (**a**) short focus and (**b**) loose focus.

Figure 6 shows the time-resolved intensities of Xe I 823.16 nm and Xe I 828.01 nm for both focusing configurations. The intensity is quantified by fitting it to the Voigt spectral line profile. The ratio of intensities of these transitions should be determined by their Einstein coefficients. If the degree of self-absorption is minimal, or approximately equal for both transitions, one would expect that the ratio of spectral line intensities would remain constant throughout the plasma lifetime. We notice the deviation from the expected intensity ratio trend in the case of loose focusing (Figure 6b). Additional work is needed to verify that the excessive broadening of the Xe I 828.01 nm spectral line is solely a result of the pronounced self-absorption. However, we also note that the increased plasma size arising from the pronounced multi-filament nature of the excitation in the Xe environment (Section 3.3) might be responsible for increased self-absorption. External focusing conditions influence the filament length and diameter, where larger filament dimensions have been shown to increase the self-absorption behavior in finely dispersed aerosols, which share similar behavior to gas species [48]. This effectively increases the optical path length traversed by the Xe emission through the colder, outer regions of the filament where the lower state population is expected to be higher, therefore resulting in pronounced self-absorption depending on the atomic transition.

### 3.3. Filament Imaging

**Figure 7:**
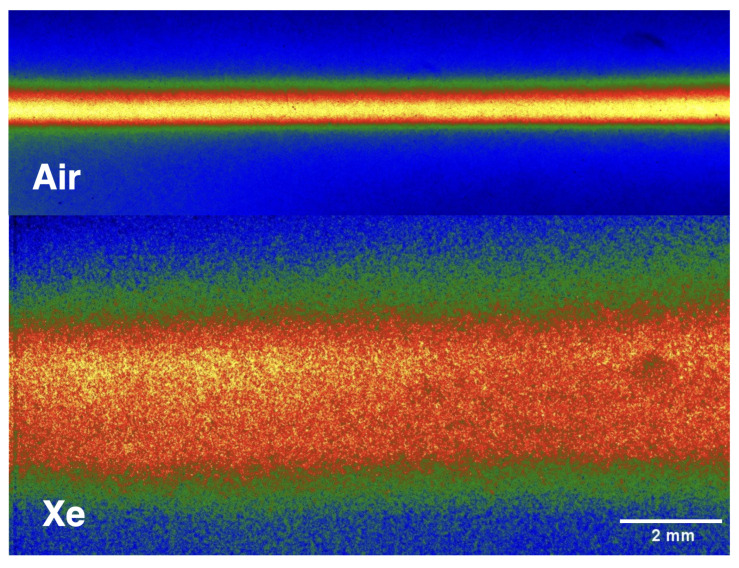
Filament images obtained under atmospheric pressure air and pure Xe ambient.

The onset of filamentation in a transparent medium depends strongly on its nonlinear refractive index [17]. Despite significant variations between the theory and experiments [49,50,51], it is commonly accepted that the $n_{2}$ values for Xe exceed the ones of air by an order of magnitude [51] at 800 nm and under atmospheric pressure, thereby lowering the critical power $P_{cr}$ needed for self-focusing. The critical power required to overcome diffraction is written as $P_{cr}=3.77\lambda^{2}/8\pi n_{2}n_{0}$ for Gaussian beams [52], where λ is the center wavelength of the laser and $n_{0}$ is the linear index of refraction. For both air and xenon $n_{0}\approx 1$, and thus changes to the critical power are driven solely by $n_{2}$. It has been estimated that $P_{cr}$ is 0.9 GW in xenon, which has been shown to improve terahertz radiation efficiency and temporal pulse compression compared to filaments generated in air [53]. However, the peak power of the laser pulses is kept consistent throughout the experiments, resulting in a higher ratio of $P/P_{cr}$ for Xe in comparison to air. Under several different focusing conditions, increasing the $P/P_{cr}$ ratio is known to increase the electron density and filament diameter [54]. When the laser power significantly exceeds $P_{cr}$, a single filament breaks up into multiple filaments such that intensity clamping occurs across each individual filament [55,56]. This multi-channel plasma structure can be seeded by non-uniformities in the beam profile. The onset and extent of multi-filamentation depend on the characteristics of the transparent medium. In an environment such as Xe, multi-filamentation is more easily initiated than in ambient air due to the lower $P_{cr}$, and, consequently, the filament breakup is more pronounced under similar experimental conditions. Given that intensity clamping implies any single filament is limited in dimension by a minimum achievable diameter, a multi-filament channel would give the appearance of an larger filament where the individual filaments cannot be spatially resolved. As a result, the multi-filamentation in Xe results in a plasma that has a larger size compared to that produced in air as evidenced in Figure 7, where individual filaments in a multi-filament channel cannot be resolved, as each image is an accumulation over 100 shots.

### 3.4. On-Axis Collection

**Figure 8:**
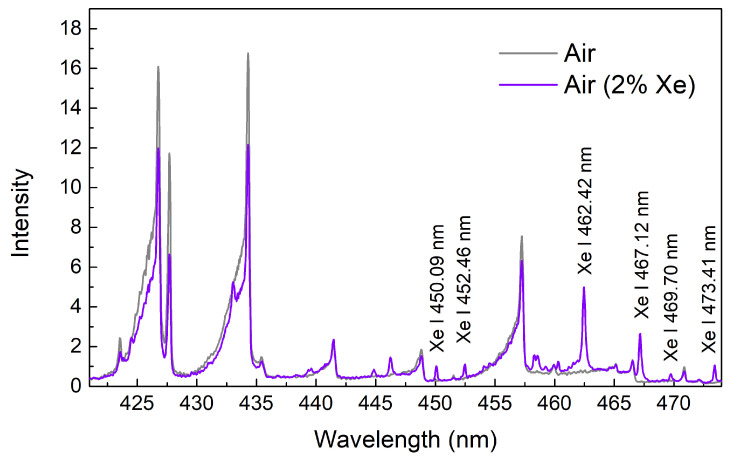
On-axis-collected loose-focus spectra of air and air–Xe mixture under atmospheric pressure.

**Figure 9:**
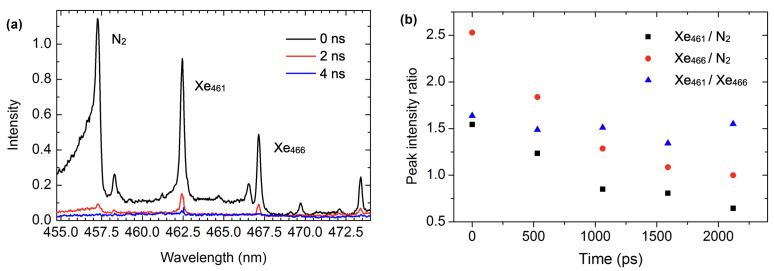
(**a**) Time-resolved, on-axis, loose-focus spectra of air–Xe mixture; (**b**) Intensity ratios of nitrogen molecular emission and Xe spectral lines.

Spectral measurements along the filamentation axis were performed solely in the VIS region with 10${}^{4}$ accumulations and a 5 ns gatewidth. The exclusive choice of the VIS spectral observation window was made due to the proximity of near-IR Xe wavelengths to the center wavelength of the laser and, therefore, poor transmission properties of the dichroic mirror in this region. Figure 8 shows the notable quenching of N${}_{2}$ molecular bands when 2% Xe is introduced into the air. The prominent Xe I transitions are present in the spectra. The Xe I spectral line at 462.42 nm appears somewhat similar to the surrounding N${}_{2}$ bands. To better distinguish atomic and molecular spectral lines, we performed time-resolved measurements with 2 ns and sub-ns resolution (Figure 9). The Xe atomic emission seems to persist longer than the neighboring molecular band. Figure 9b shows the exponentially decaying intensity ratios of the peak intensities of atomic and molecular transitions as well as the approximately constant intensity ratio of Xe transitions. This clearly shows the difference between transitions of atomic emission versus the emission of molecular origin. Future research directions should focus on the detection of trace (parts per million) amounts of Xe in the form of calibrated air–Xe mixtures as well as extending this approach to the near-IR region. In the on-axis collection configuration, the challenge with the latter improvement can be addressed by developing a custom-coated optic with a sharp wavelength cutoff to transmit the spectral region around 823 nm while reflecting the entire laser bandwidth. Alternatively, the prominent Xe I 881.94 nm transition can be investigated given the favorable sensitivity of the detector.

It is important to distinguish two sources of continuum radiation related to the measurements of filament-induced plasma emission. The first source is dominantly governed by radiative electron-ion recombination and inverse *bremsstrahlung* within the formed plasma. As seen in Section 3.1, this continuum emission persists from hundreds of ns for short-focus configuration to several ns in the case of filament excitation. On the other hand, the femtosecond pulse is accompanied by supercontinuum light production through self-phase modulation (SPM). We note that our observations are performed in conjunction with the laser (and most of the supercontinuum) propagation direction. If the scattered SPM supercontinuum presents an issue, one might also consider employing the polarization gating technique [57] to minimize potential interference with the analyte signal. The placement of a polarizer before the detector can filter out the filament-induced supercontinuum background in the spectra of filament-induced breakdown.

## 4. Conclusions

The detection of Xe in the atmosphere holds significance for a range of purposes, predominantly within the realms of environmental surveillance, nuclear safety, and scientific investigation. Xe is generated as a by-product of nuclear fission within nuclear reactors. The importance of monitoring and detecting xenon levels in the vicinity of nuclear facilities is underscored by the imperatives of safety and regulatory adherence. Unexpected surges in xenon concentrations can serve as an early warning sign of possible problems or leaks within the reactor, thus contributing to accident prevention and the maintenance of facility integrity. Although regarded as non-toxic, elevated concentrations of Xe can serve as an indicator of potential issues, such as the release of radioactive materials stemming from a nuclear incident or clandestine underground nuclear tests. The monitoring of Xe levels in the atmosphere plays a crucial role in the global initiatives aimed at detecting and upholding the Comprehensive Nuclear Test Ban Treaty. As an alternative to techniques dependent on radioactive decay, LIBS stands out as a promising technique to produce distinctive optical characteristics specific to Xe. Compared to nanosecond and short-focus femtosecond-induced excitation, Xe transitions with upper-level energy exceeding 10 eV are still present, but they are expectedly weaker in filament-induced excitation. Nevertheless, the most prominent spectral signatures are still present in the filament spectra despite several orders of magnitude differences in laser intensity compared to the short-focus excitation. Both focusing geometries were investigated by observing the spectral regions in the VIS and near-IR ranges. The presence of prominent transitions in the filament-excitation case was further verified when the amount of Xe sample was reduced 50× in the air–Xe mixture. Research directions that focus on the detection of ppm amounts of Xe in calibrated air–Xe mixtures, along with the extension of this approach to the near-IR region, have the potential to contribute significantly to nuclear applications and environmental monitoring.

## Data Availability

The data presented in this study are available on request from the corresponding author.
